# Exogenous melatonin increases salt tolerance in bitter melon by regulating ionic balance, antioxidant system and secondary metabolism-related genes

**DOI:** 10.1186/s12870-022-03728-0

**Published:** 2022-07-30

**Authors:** Morteza Sheikhalipour, Seyed Abolghasem Mohammadi, Behrooz Esmaielpour, Elnaz Zareei, Muhittin Kulak, Sajid Ali, Mojtaba Nouraein, Mohammad Kazem Bahrami, Gholamreza Gohari, Vasileios Fotopoulos

**Affiliations:** 1 Department of Horticulture, Faculty of Horticulture, University of Mohagheh Ardebili, Ardebil, Iran; 2 Department of Plant Breeding and Biotechnology, Faculty of Agriculture, University of Tabriz, Tabriz, Iran; 3 Center for Cell Pathology, Department of Life Sciences, Khazar University, Baku, Azerbaijan; 4 Department of Horticultural Science, Faculty of Agriculture, University of Kurdistan, Sanandaj, Iran; 5 Department of Herbal and Animal Production, Vocational School of Technical Sciences, Igdir University, Igdir, Türkiye; 6 Department of Horticulture, Bahauddin Zakariya University, Multan, Punjab, Pakistan; 7 Department of Plant Genetics and Production, Faculty of Agriculture, University of Maragheh, Maragheh, Iran; 8 Department of Biology, Faculty of Sciences, University of Maragheh, Maragheh, Iran; 9 Department of Horticultural Science, Faculty of Agriculture, University of Maragheh, Maragheh, Iran; 10 Department of Agricultural Sciences, Biotechnology and Food Science, Cyprus University of Technology Limassol, Limassol, Cyprus

**Keywords:** *Momordica charantia* L., Abiotic stress, Salinity, Defense transcripts

## Abstract

**Background:**

Melatonin is a multi-functional molecule widely employed in order to mitigate abiotic stress factors, in general and salt stress in particular. Even though previous reports revealed that melatonin could exhibit roles in promoting seed germination and protecting plants during various developmental stages of several plant species under salt stress, no reports are available with respect to the regulatory acts of melatonin on the physiological and biochemical status as well as the expression levels of defense- and secondary metabolism-related related transcripts in bitter melon subjected to the salt stress.

**Results:**

Herewith the present study, we performed a comprehensive analysis of the physiological and ion balance, antioxidant system, as well as transcript analysis of defense-related genes (*WRKY1*, *SOS1, PM H*^*+*^*-ATPase, SKOR, Mc5PTase7*, and *SOAR1*) and secondary metabolism-related gene expression (*MAP30, α-MMC*, *polypeptide-P*, and *PAL*) in salt-stressed bitter melon (*Momordica charantia* L.) plants in response to melatonin treatment. In this regard, different levels of melatonin (0, 75 and 150 µM) were applied to mitigate salinity stress (0, 50 and 100 mM NaCl) in bitter melon. Accordingly, present findings revealed that 100 mM salinity stress decreased growth and photosynthesis parameters (SPAD, ^Fv^/_Fo_, Y(II)), RWC, and some nutrient elements (K^+^, Ca^2+^, and P), while it increased Y(NO), Y(NPQ), proline, Na^+^, Cl^−^, H_2_O_2_, MDA, antioxidant enzyme activity, and lead to the induction of the examined genes. However, prsiming with 150 µM melatonin increased SPAD, ^Fv^/_Fo_, Y(II)), RWC, and K^+^, Ca^2+^, and P concentration while decreased Y(NO), Y(NPQ), Na^+^, Cl^−^, H_2_O_2_, and MDA under salt stress. In addition, the antioxidant system and gene expression levels were increased by melatonin (150 µM).

**Conclusions:**

Overall, it can be postulated that the application of melatonin (150 µM) has effective roles in alleviating the adverse impacts of salinity through critical modifications in plant metabolism.

**Supplementary Information:**

The online version contains supplementary material available at 10.1186/s12870-022-03728-0.

## Background

Salinity is of the major constraints affecting world agricultural production, appearing as one of the major challenges to be alleviated because of its retarding effects on growth, development and productivity of crops [1, 2]. Nearly 50% of irrigated land and 10% of soils in the world are under exposure to high levels of salinity [3]. Due to their sessile nature, plants cannot escape from the environmental cues and for that reason, they have to evolve an elaborate system as well as adaptive responses against salt stress. Corresponding to the high levels of salinity, the sodium and chloride ions accumulate in the soil, which in turn reduces the availability of essential nutrients (such as K^+^) and water in plants [4]. K^+^/Na^+^ homeostasis is one of the key mechanisms for salinity tolerance in plants and in this regard, regulation/compartmentalization of Na^+^ and K^+^ homeostasis in plants is critical for enhanced salt stress tolerance [5]. In cytosol, plasma membrane Na^+^/H^+^ antiporter (SOS1), SKOR K^+^ channel, and the PM H^+^-ATPase regulate Na^+^/K^+^ ion homeostasis under salinity stress. Furthermore, reactive oxygen species (ROS) signaling exhibits a crucial role linked to salinity tolerance [6]. Nicotinamide adenine dinucleotide phosphate (NADPH) oxidase is the main source of apoplastic ROS production which leads to salinity tolerance in the various plant [7]. In addition, gene family of WRKY, a plant-specific transcriptions factor (TF) group, plays key functions in various response pathways. For example, WRKY1 is involved in plant tolerance against drought [8] and salinity [9]. SOAR1, a cytosolic-nuclear pentatricopeptide repeat protein, has a vital role in plant response to salinity and drought [10, 11]. Furthermore, phenylalanine ammonia-lyase (PAL) is a well-known precursor to increase production of major secondary metabolites which are, in general, crucial for plant adaptation against biotic and abiotic stress factors [12, 13].

Melatonin (MT) has been shown to have various potential physiological functions in plants under stressful and non-stressful conditions [14]. Among the known functions, MT has been revealed to be effective in alleviating the oxidative damage of stress factors, viz. heavy metal [14], high temperature [15], salt [16], drought [17] and cold stress [18]. In this context, Zhang and Zhang [19] and Reiter et al. [20] reported that MT is a mitochondria-targeted antioxidant that achieves this action directly (detoxification of RONS) or indirectly (by inducing antioxidant enzymatic activity and suppressing pro-oxidant enzymatic activity). It has also been reported that MT as a regulator of growth and/or biostimulator in plants [21, 22] and could be effective in triggering germination by biosynthesis regulation and catabolism GA4 and ABA in cucumbers [23], stimulating development of roots owing to the regulation of auxins synthesis, signaling and transport, as observed in tomatoes and Arabidopsis [24], increasing berry quality of grape [25] and improving the postharvest conservation of fresh fruits and vegetables [26]. Moreover, exogenous applications of MT increased the secondary metabolite contents in cabbage plants via up-regulating the expression of related biosynthetic genes [27].

*Momordica charantia* L., commonly known as bitter melon or bitter gourd is an important member of family cucurbitaceae and it widely grows in tropical and sub-tropical areas. The fruit and leaves of bitter melon are rich in phytochemicals including nutraceutical and nutritional components [28]. Bitter melon has a wide range of medical applications to treat cancer, hypertension, T2DM, bacterial and viral infections, obesity, and even AIDS [29]. Anti-HIV protein, MAP30, and α-momorcharin (α-MMC) are Type-I RIPs (Ribosome-Inactivating Proteins) having single enzyme chain, which was isolated from bitter melon and demonstrated to have efficacy against HIV infection and cancer. Polypeptide-P, another bioactive peptide isolated from bitter melon, showed hypoglycemic activity in diabetes [30].

The excellent functions of MT as anti-stressor have been widely reported for several crops. For instance, MT critically altered plant responses against stress through reducing the levels of H_2_O_2_, activating the ROS-metabolizing enzymes and inducing Na^+^ and K^+^ transporters. Those modifications assisted in alleviating the adverse effects of salinity [21, 31]. Interestingly, it has been surmised that melatonin produce minor metabolites, which then co-work in combating with the stress. For that reason, Back [32] hypothesized that melatonin and its metabolites are together in the case exogenous applications of melatonin, suggesting that marked responses of the plants cannot be exclusively attributed to the melatonin alone. For that reason, the studies linked to reveal the roles of MT are required. In this regard, we herein carried a comprehensive study in bitter melon subjected to salt stress. The regulatory roles of MT on the expression levels of defense- and secondary metabolism-related related transcripts in bitter melon subjected to the salt stress were, for the first time, investigated. The key objectives of the current work were: (i) to study the potential function of MT in ameliorating the negative effect of salinity, (ii) to decipher the expression profile of defense and secondary metabolism-related genes induced by MT under salt stress, and (iii) to examine the physiological, biochemical, and nutritional state of MT-primed plants under salt stress, mainly aiming to investigate molecular regulatory components involved in the response to salinity.

## Materials and methods

### Growth conditions and plant treatment

This experiment was conducted in a growth chamber of the Department of Plant Biotechnology, University of Tabriz. The experiment was performed as a factorial using a completely randomized design (CRD) with three biological replications and each replication consisted of two plants. The bitter melon (Palee F1) seeds were provided from Victoria Companies, India. Seeds were sterilized using sodium hypochlorite solution (1%) for 5 min and were then left for germination under darkness at 25 °C for 48 h for pre-germination. Following one-week germination, the seeds those were homogeneously and uniformly germinated were transferred to the trays for cultivation comprising coco-peat and watered with ½ Hoagland’s modified solution. The seeds containing trays were then placed in the growth chamber with 28/22°C (day/night) and relative humidity of 62–80% 360 µmol m^− 2^ s^− 1^ light intensity. At the time of third true leaf emergence, healthy and uniform seedlings were selected and separated into three groups: (i) Hoagland’s nutrient solution (HNS, as a control); (ii) HNS + 75 µM melatonin (MT); and (iii) HNS + 150 µM MT. The concentrations of melatonin were selected according to Yin et al. [33]. Plants were grown in melatonin-supplemented Hoagland solution for one week with 0, 75 and 150µM MT (MT added to Hoagland’s nutrient solution). MT was dissolved in ethanol were then diluated with MilliQ water. The MT purchased from Sigma–Aldrich, company, USA. Four days after MT supplementation, plants were exposed to different levels of salinity stress (0, 50 and 100 mM NaCl) for three day period. After treatment, leaves and roots samples were harvested for RNA extraction and study of gene expression, frozen in liquid nitrogen promptly after harvest, and stored at ˗80 °C until further analyses.

### Growth parameters

Fresh and dry weights of shoot and root, as well as height of shoot and root, were measured at the end of three-day salt stress.

### Chlorophyll-related parameters

The chlorophyll index in five fully-expanded leaves was recorded with a SPAD-502 chlorophyll meter (Minolta Co. Ltd., Japan). Chlorophyll fluorescence parameters (^Fv^/_Fo_, Y(II), Y(NO) and Y(NPQ)) of leaf samples were assessed with a chlorophyll fluorometer (Dual-PAM-100, Heinz Walz, Efeltrich, Germany) under dark adaption for 20 min.

### Relative permeability, proline contents and relative water content

Relative permeability was determined according to the method of Nanjo et al. [34]. Proline content in leaf samples was determined with detailed method of Bates et al. [35]. Freshly sourced leaves were macerated in 3% sulphosalicylic acid solution and were then centrifuged for 10 min at 15,000 × g at 4 °C. One ml of prepared supernatant solution was placed in a tube and reacted with one ml acid ninhdrin and one ml of acetic acid. The resultant mixtures were heated for 60 min at 100^º^C. The assay reaction was stopped by putting the reaction assay on ice. After that, toluene (2 mL) was used for the extraction of assay mixture. The two phases were separated by keeping the reaction assay at room temperature for the period of 30 min. Finally, supernatants absorbance was read at 520 nm on a spectrophotometer (UV-1800 Shimadzu, Japan) and toluene was served as blank. The relative water content (RWC) of leaf samples was measured following the protocol described by Sairam and Srivastava [36]. Initial fresh leaf samples were weighted using digital balance for fresh weight (FW). Then, turgid weight (TW) was recorded by placing the leaf samples in double-distilled water for 24 h. Finally, dry weights (DW) of samples were assessed after 24 h drying at 70 ºC. RWC was measured by the equation: RWC= (FW − DW)/(TW − DW)×100.

### Malondialdehyde (MDA) and hydrogen peroxide (H_2_O_2_) content

The concentration of malondialdehyde (MDA) was determined with previous protocol [37]. The 0.3 g fresh leaf sample was ground in 20% trichloroacetic acid (TCA) and centrifuged for 15 min at 13,000×. Thereafter, TCA (20%, 4 mL) was incorporated into 1 ml of the solution of supernatant. The mixture was the boiled in water bath (95 °C) for 30 min. Afterward, the mixture was quaickly cooled in an ice bath and absorbance was noted at 600 nm and 532 nm. Finally, MDA was calculated by used 155 mm^− 1^ cm^− 1^ as a coefficient of molar absorption.

H_2_O_2_ was determined with protocol of Allen [38]. Briefly, 0.2 g sample of the leaves was macerated in an ice bath which contained 0.1% TCA (3 mL) and it was centrifuged for 15 min at 20,000×g. After that, 500 µL assay mixture was reacted with 10 mM concentrated phosphate buffer (500 µL, 7.0 pH) comprising 2 M KI. The resultant assay was kept under darkness for 60 min at room temperature for the incubation. Finally, H_2_O_2_ content was assayed at 390 nm on a spectrophotometer.

### Antioxidant enzymes activities

In order to assay the activity of antioxidant enzymes, 0.5 g homogenized leaf sample was macerated in 0.05 M phosphate buffer (1% PVP, 1 M MEDTA, 7.8 pH), and subjected to centrifugation for 20 min at 12,000× g (4 °C). The collected supernatants were used for peroxidase (POD) [39] and superoxide dismutase (SOD) activities determination [40]. To determine POD activity, assay reaction comprising enzymes extract, 5 µL of 10% (w/v) H_2_O_2_, 100 mM phosphate buffer (pH 6.0), and 16 mM guaiacol. The absorbance was at 470 nm for 1 min as mmol produced tetraguaiacol per minute per mg soluble proteins (U mg^− 1^). As well as one unit of SOD activity was enzyme amount needed to cause 50% inhibition of NBT (nitro blue tetrazolium) at 560 nm.

### Content of nutrient elements

For quantifying the content of major macro-elements, 100 mg of ground, oven-dried root tissue was digested in concentrated nitric acid (110 °C for 6 h). The concentration of potassium (K^+^) and sodium (Na^+^) in the digested extracts was quantified by flame emission spectrometry, while calcium (Ca^2+^) and phosphorus (P) were determined by atomic absorption spectrometry (AA-7000, Shimadzu). For chloride (Cl^−^) determination, oven-dried root samples were extracted with deionized water at 100 °C for 2 h, after which Cl^−^ content was measured by ion chromatography (ICS 2000, Dionex, Sunnyvale, CA, USA).

### RNA isolation and quantitative real-time PCR (RT-qPCR) assay

Total RNA extracted from the leaf and root samples (using CinnaGen kit, Iran) was used for cDNA synthesis (using Yekta Tajhiz Azma kit, Iran). The primers for the *α-tubulin 1a* internal control gene and studied genes (*MAP30, α-MMC, polypeptide-P, SOS1, H*^*+*^*-ATPase, SKOR, SOAR1, Mc5PTase7*, and *WRKY1*) are shown in Table S1. The RT-qPCR reaction mixtures (25 µL) contained 12.5 µL of master mix (AMPLIQON), 1 µL of primer (10 µM), 2 µL of cDNA, and 9.5 µL of nuclease-free water. The reaction parameters were used in all cycle sequencing reactions: initial denaturation at 95 °C for 30 s; denaturation at 95 °C for 5 s, annealing at 60 °C for 20 s, 30–40 cycles; 55° to 95 °C increased by 0.5 °C every 30 s, 81 cycles. Three replicates were calculated for each sample and gene the relative expression of the gene was calculated by the comparative Ct (2^−ΔΔCt^) method.

### Statistical analysis

The data obtained were analyzed by Statistica-13 (Statsoft, Tulsa, USA). Factorial ANOVA, in which the concentration of MT solution and degree of salinity stress were used as categorical variables revealed a significant difference between treatments. When a significant difference was found, Duncan’s post-hoc analysis was used to find homogeneous groups (p < 0.05, significant difference).

## Results

### Effect of exogenous melatonin on growth parameters under salinity stress condition

To assess the effects of MT and salt stress in bitter melon, four-week-old bitter melon seedlings were subjected to MT pre-treatment, and were then treated with 50 and 100 mM NaCl stress for three days. In relation to the control, the higher levels of salinity critically decreased shoot height, root height, shoot fresh weight, shoot dry weight, root fresh weight, and root dry weight up to 51.25%, 57.23%, 48.51%, 48.46%, 42.18%, and 42.01% respectively (*p* < 0.05) (Table 1). As expected, 150 µM concentration of MT substantially increased shoot height (15.04% and 30.46%), root height (19.23% and 37.43%), shoot fresh weight (20.18% and 22.15%), shoot dry weight (20.07% and 21.76%), root fresh weight (14.58% and 20.68%) and root dry weight (14.74% and 15.04%) than salt-treated biter melons with 50 and 100 mM (*p* < 0.05) (Table 1).

**Table 1:** Effect of application of melatonin (0, 75 and 150 µM) on growth parameters of bitter melon (*Momordica charantia*) under salt stress (0, 50 and 100 mM NaCl) conditions

NaCl (mM)	Melatonin (μM)	Shoot length (cm)	Root length (cm)	Shoot FW (g)	Shoot DW (g)	Root FW (g)	Root DW (g)
**0**	**0**	19.57 ± 0.62 ^a^	14.03 ± 0.49^a^	33.31 ± 0.40^b^	2.93 ± 0.03^b^	0.422 ± 0.016^ab^	0.0238 ± 0.0009^ab^
	**75**	19.76 ± 0.42 ^a^	14.42 ± 0.50^a^	34.02 ± 0.06^ab^	2.99 ± 0.00^ab^	0.424 ± 0.013^ab^	0.0240 ± 0.0007^ab^
	**150**	20.08 ± 0.40 ^a^	14.61 ± 0.45^a^	34.66 ± 0.48^a^	3.05 ± 0.04^a^	0.433 ± 0.013^a^	0.0245 ± 0.0007^a^
**50**	**0**	15.59 ± 0.37 ^d^	10.92 ± 0.50^c^	25.35 ± 0.99^e^	2.23 ± 0.08^e^	0.328 ± 0.014^de^	0.0185 ± 0.0008^de^
	**75**	16.60 ± 0.33 ^c^	12.09 ± 0.50^b^	28.76 ± 0.49^d^	2.53 ± 0.04^d^	0.359 ± 0.011^cd^	0.0203 ± 0.0006^cd^
	**150**	18.35 ± 0.19 ^b^	13.52 ± 0.50^a^	31.76 ± 0.36^c^	2.79 ± 0.03^c^	0.384 ± 0.011^bc^	0.0217 ± 0.0006^bc^
**100**	**0**	9.54 ± 0.13 ^g^	6.00 ± 0.51^f^	17.15 ± 0.25^g^	1.51 ± 0.02^g^	0.244 ± 0.010^f^	0.0138 ± 0.0006^f^
	**75**	11.56 ± 0.13 ^f^	7.92 ± 0.48^e^	21.02 ± 0.45^f^	1.85 ± 0.03^f^	0.290 ± 0.045^e^	0.0164 ± 0.0025^e^
	**150**	13.72 ± 0.19 ^e^	9.59 ± 0.46^d^	22.03 ± 0.12^f^	1.93 ± 0.01^f^	0.308 ± 0.011^e^	0.0174 ± 0.0006^e^

^a-h^show significant difference according to Duncan’s multiple range test at *p* ≤ 0.05

### Effect of exogenous melatonin on photosynthetic parameters under salinity stress condition

High concentration of salt significantly reduced SPAD, ^Fv^/_Fo_ and Y (II) and increased Y (NO) and Y (NPQ), in comparison to the control (Table 2). Being consistent with the former reports [41], MT (150 µM) reduced the adverse impacts of high level of salinity by increasing the values of SPAD, ^Fv^/_Fo_, Y(II) and by decreasing Y(NO) and Y(NPQ), in comparison to either unprimed or salt-stressed plants (*p* < 0.05).

**Table 2:** Effect of application of melatonin (0, 75 and 150 µM) on photosynthetic parameters of bitter melon (*Momordica charantia*) under salt stress (0, 50 and 100 mM NaCl) conditions

NaCl (mM)	Melatonin (μM)	SPAD	F_v_/F_o_	Y (II)	Y (NO)	Y (NPQ)
**0**	**0**	38.15 ± 0.76^a^	2.91 ± 0.02^b^	0.590 ± 0.002^a^	0.289 ± 0.004^g^	0.183 ± 0.003^f^
	**75**	37.80 ± 1.02^a^	2.96 ± 0.01^b^	0.590 ± 0.006^a^	0.284 ± 0.005^g^	0.179 ± 0.003^f^
	**150**	39.02 ± 1.07^a^	3.05 ± 0.04^a^	0.594 ± 0.005^a^	0.278 ± 0.004^g^	0.171 ± 0.001^g^
**50**	**0**	32.2 ± 0.68^c^	2.31 ± 0.03^e^	0.431 ± 0.002^d^	0.395 ± 0.011^d^	0.233 ± 0.004^c^
	**75**	34.2 ± 0.71^b^	2.48 ± 0.02^d^	0.477 ± 0.006^c^	0.352 ± 0.004^e^	0.214 ± 0.004^d^
	**150**	35.57 ± 0.48^b^	2.78 ± 0.02^c^	0.516 ± 0.012^b^	0.326 ± 0.009^f^	0.200 ± 0.004^e^
**100**	**0**	28.59 ± 0.77^d^	1.66 ± 0.06^h^	0.264 ± 0.004^g^	0.595 ± 0.005^a^	0.260 ± 0.003^a^
	**75**	30.84 ± 0.66^c^	1.77 ± 0.02^g^	0.301 ± 0.010^f^	0.545 ± 0.011^b^	0.253 ± 0.001^a^
	**150**	31.69 ± 1.07^c^	1.91 ± 0.05^f^	0.345 ± 0.008^e^	0.505 ± 0.007^c^	0.242 ± 0.002^b^

^a-h^show significant difference according to Duncan’s multiple range test at *p* ≤ 0.05

### Effect of exogenous melatonin on proline and RWC under salinity stress condition

**Fig. 1:**
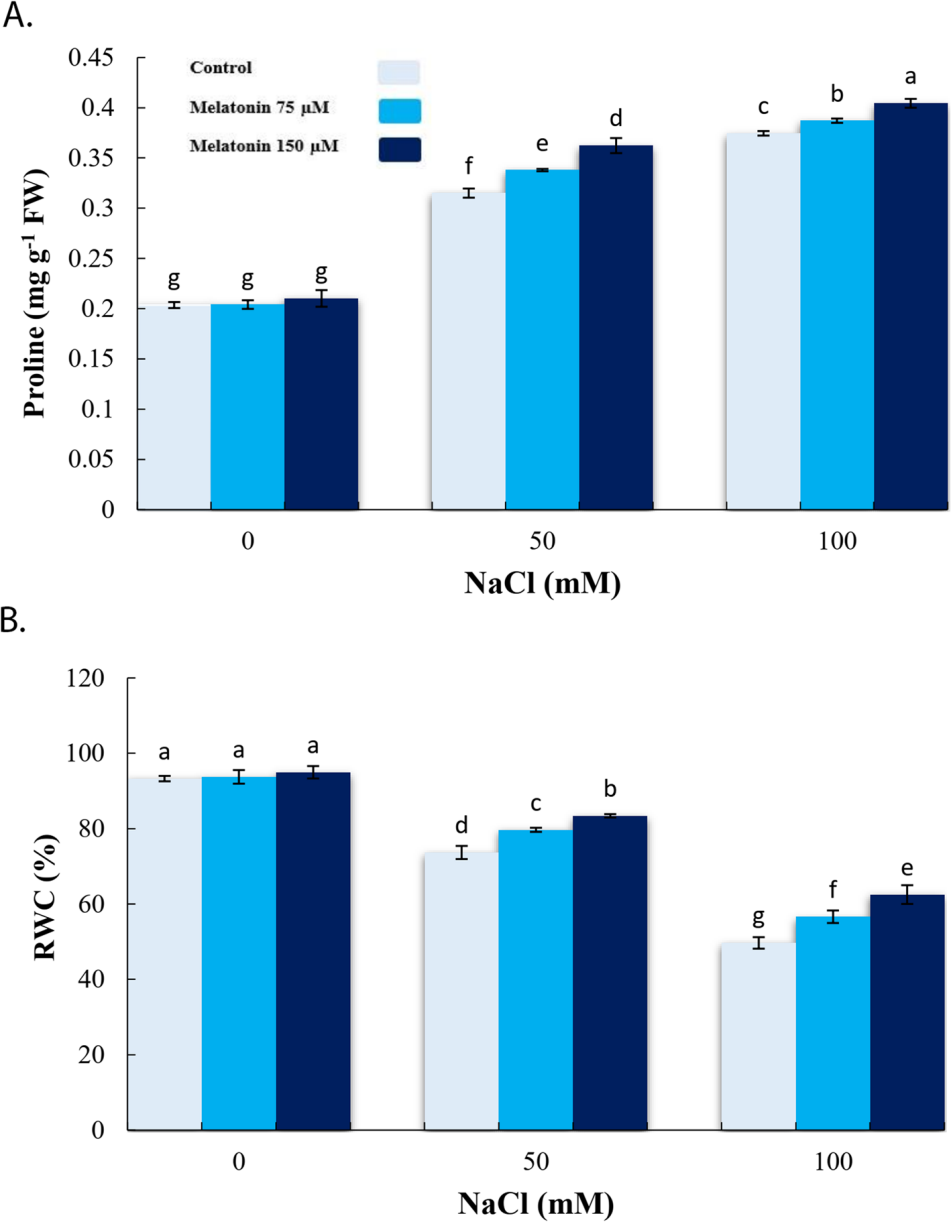
Effect of application of melatonin (0, 75 and 150µM) on Proline (**a**) and RWC (**b**) in bitter melon under salt stress (0, 50 and 100 mM NaCl) conditions. Data are the average of 3 replicas ± standard error. Different letters show significant difference according to Duncan’s multiple range test at *p* ≤ 0.05

High concentration of salt stress caused significant increments in proline content, while it decreased RWC. Once compared with 50 and 100 mM NaCl stress, pre-treatments with MT (150 µM) increased proline and RWC (*p* < 0.05) (Fig. 1a, b).

### Effect of exogenous melatonin on oxidative stress indicators under salinity stress condition

**Fig. 2:**
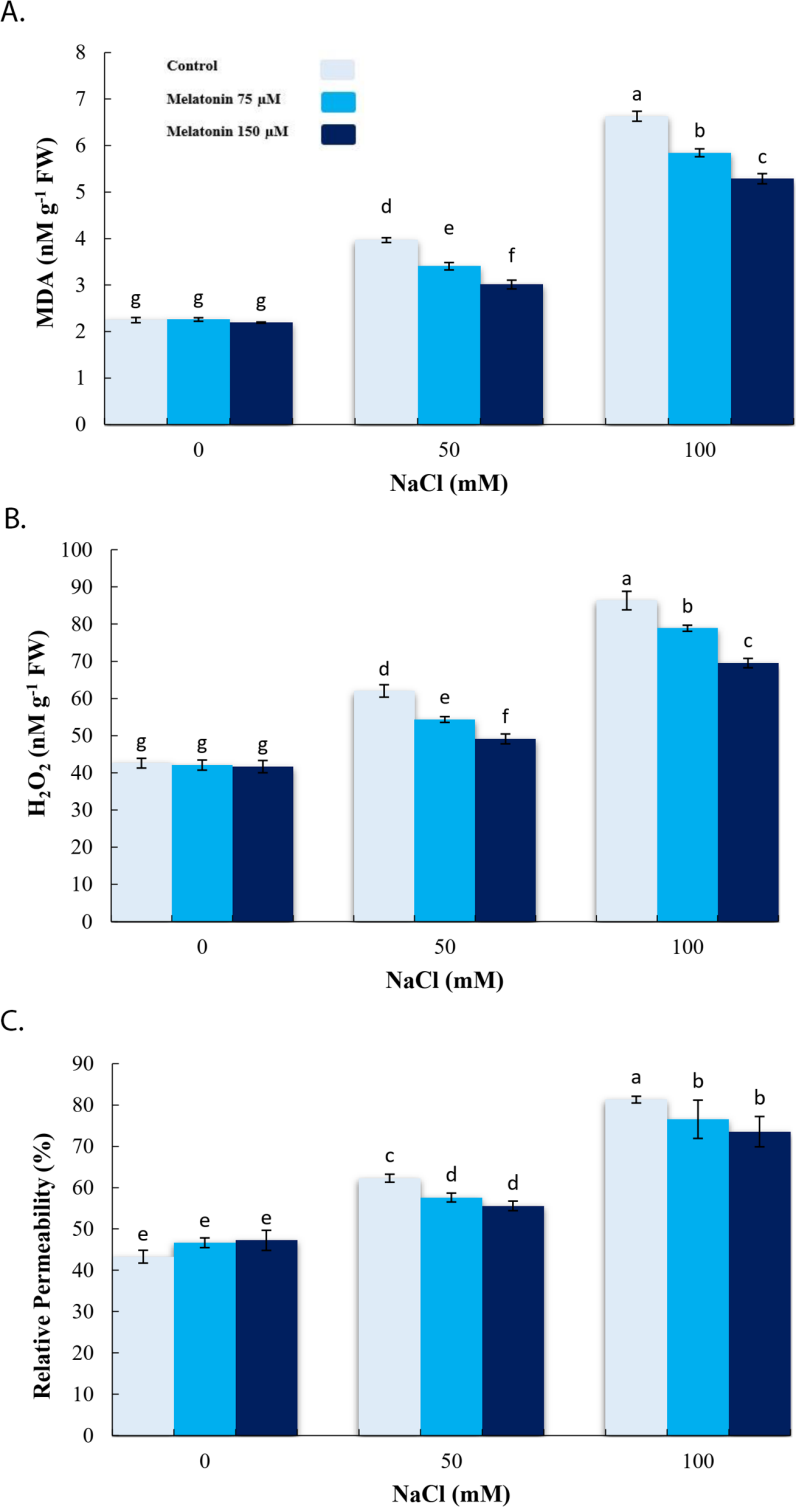
Effect of application of melatonin (0, 75 and 150µM) on MDA (**a**), H_2_O_2_ (**b**) and Relative Permeability (%) (**c**) content in bitter melon under salt stress (0, 50 and 100 mM NaCl) conditions. Data are the average of 3 replicas ± standard error. Different letters show significant difference according to Duncan’s multiple range test at *p* ≤ 0.05

In regard to common cellular damage indicators, 100 mM NaCl increased the level of MDA, H_2_O_2_ and electrolyte leakage, in comparison to the control. Along with the pre-treatment of 150 µM MT, significant reductions were observed in levels of MDA, H_2_O_2_ and electrolyte leakage, in comparison to either the unprimed or 50 and 100 mM NaCl treatments (Fig. 2a-c).

### Effect of exogenous melatonin on antioxidative enzymes activities under salinity stress condition

**Fig. 3:**
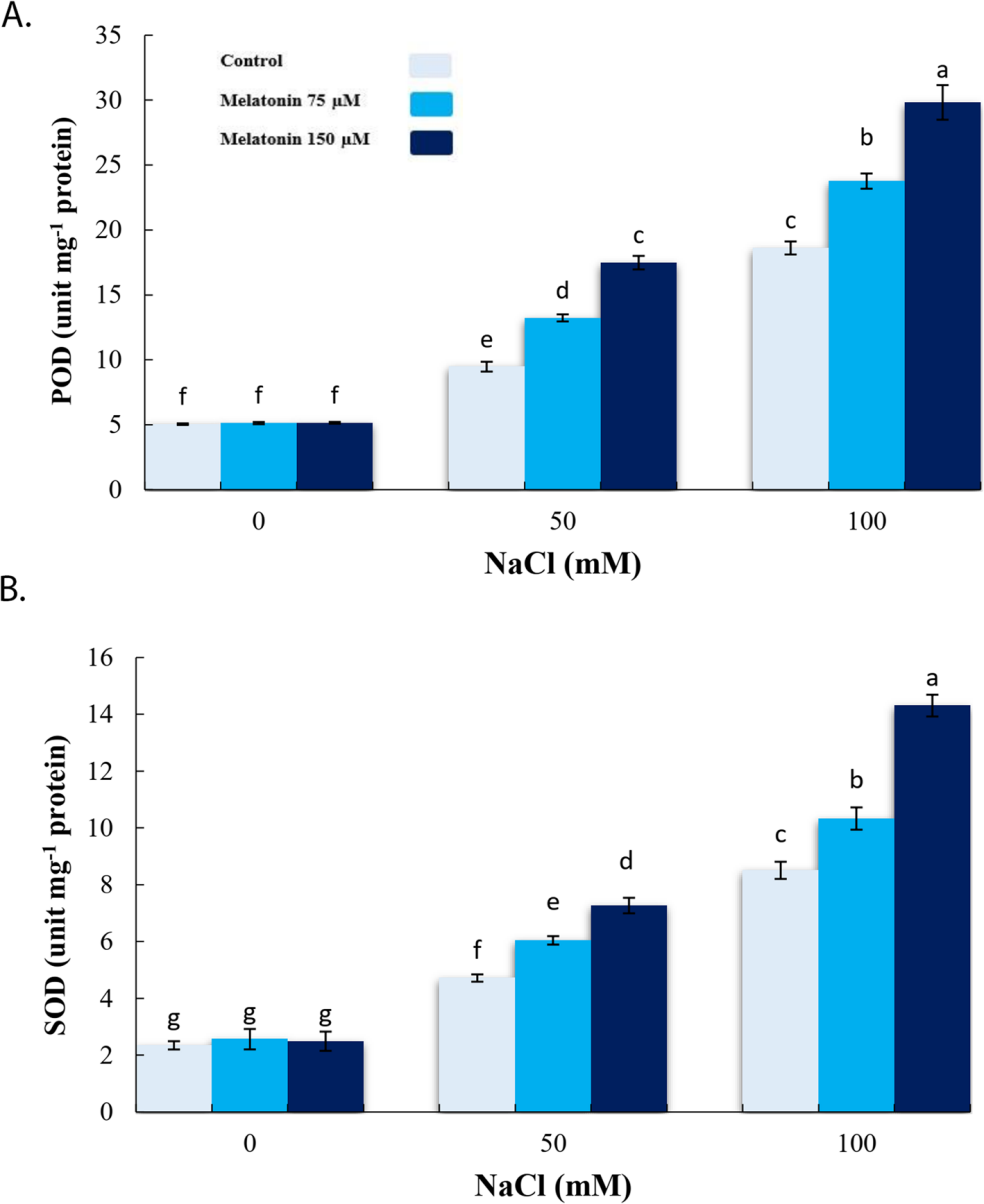
Effect of application of melatonin (0, 75 and 150µM) on POD (**a**) and SOD (**b**) enzyme activity in bitter melon under salt stress (0, 50 and 100 mM NaCl) conditions. Data are the average of 3 replicas ± standard error. Different letters show significant difference according to Duncan’s multiple range test at *p* ≤ 0.05

In accordance with the increased stress-related parameters, significant increases in activities of POD and SOD were observed at 100 mM NaCl, relative to the control. However, pretreatment with 150 µM MT further increased the activity of POD and SOD more than salinity alone, in comparison with 50 and 100 mM NaCl (Fig. 3a, b).

### Effect of exogenous melatonin on nutrient concentration under salinity stress condition

**Fig. 4:**
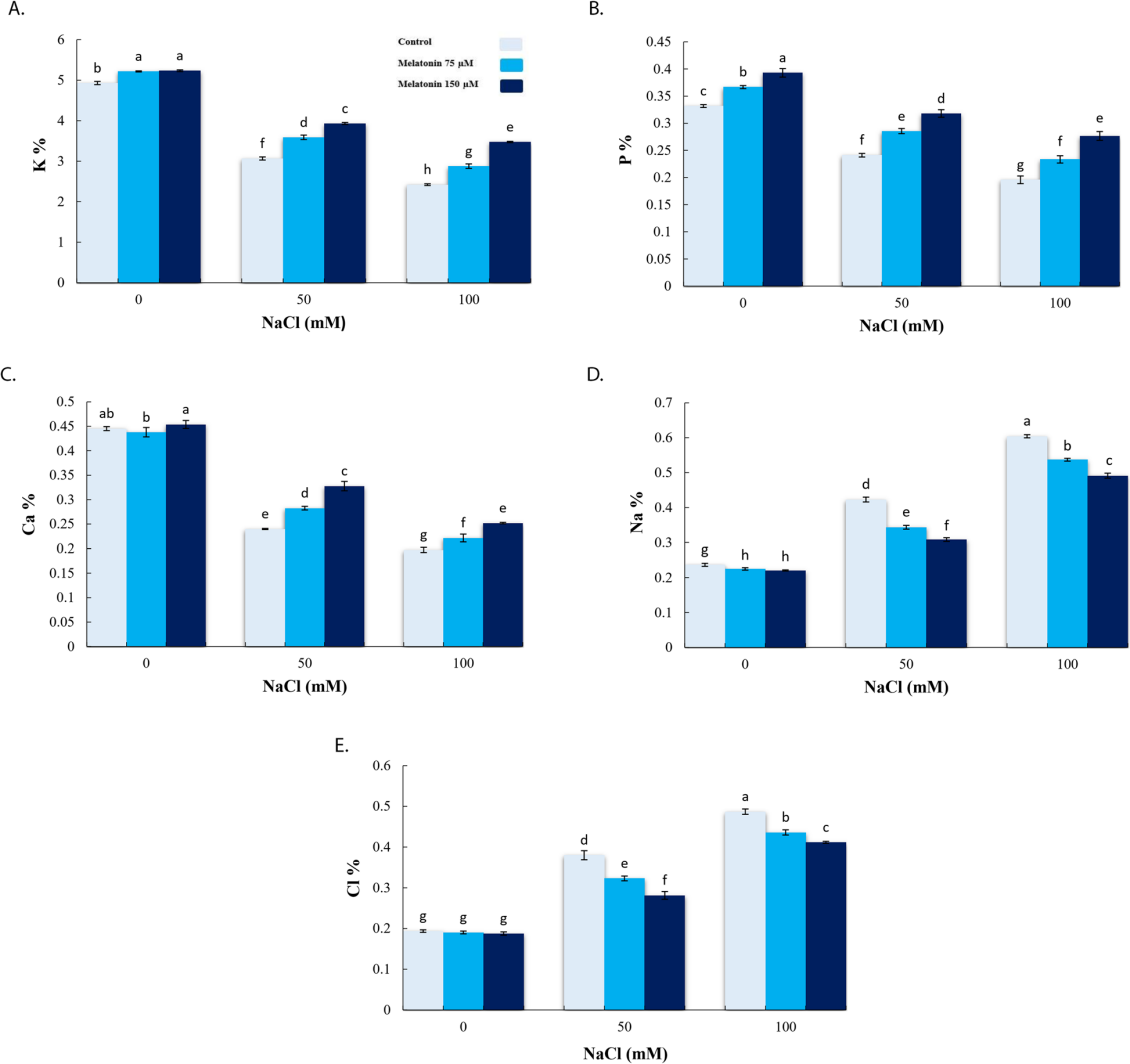
Effect of application of melatonin (0, 75 and 150µM) on K^+^ (**a**), P (**b**), Ca^2+^ (**c**), Na^+^ (**d**) and Cl^−^ (**e**) content of bitter melon roots under salt stress (0, 50 and 100 mM NaCl) conditions. Data are the average of 3 replicas ± standard error. Different letters show significant difference according to Duncan’s multiple range test at *p* ≤ 0.05

In relation to the control, sharp decreases in K^+^, P and Ca^2+^ content by 50.88%, 40.97%, and 55.69%, respectively, and increases in Na^+^ and Cl^−^ of up to 60.83% and 60.22% were observed in roots under 100 mM NaCl. However, pretreatment with 150 µM MT increased K^+^ (21.98% and 30.29%), P (24.18% and 29.21%) and Ca^2+^ (26.77% and 21.67%) content and decreased Na^+^ (27.07% and 18.68%) and Cl^−^ (25.97% and 15.46%) content, in relation to 50 and 100 mM NaCl (Fig. 4a-e).

### Effect of exogenous melatonin on defense-related genes expression under salinity stress conditions

**Fig. 5:**
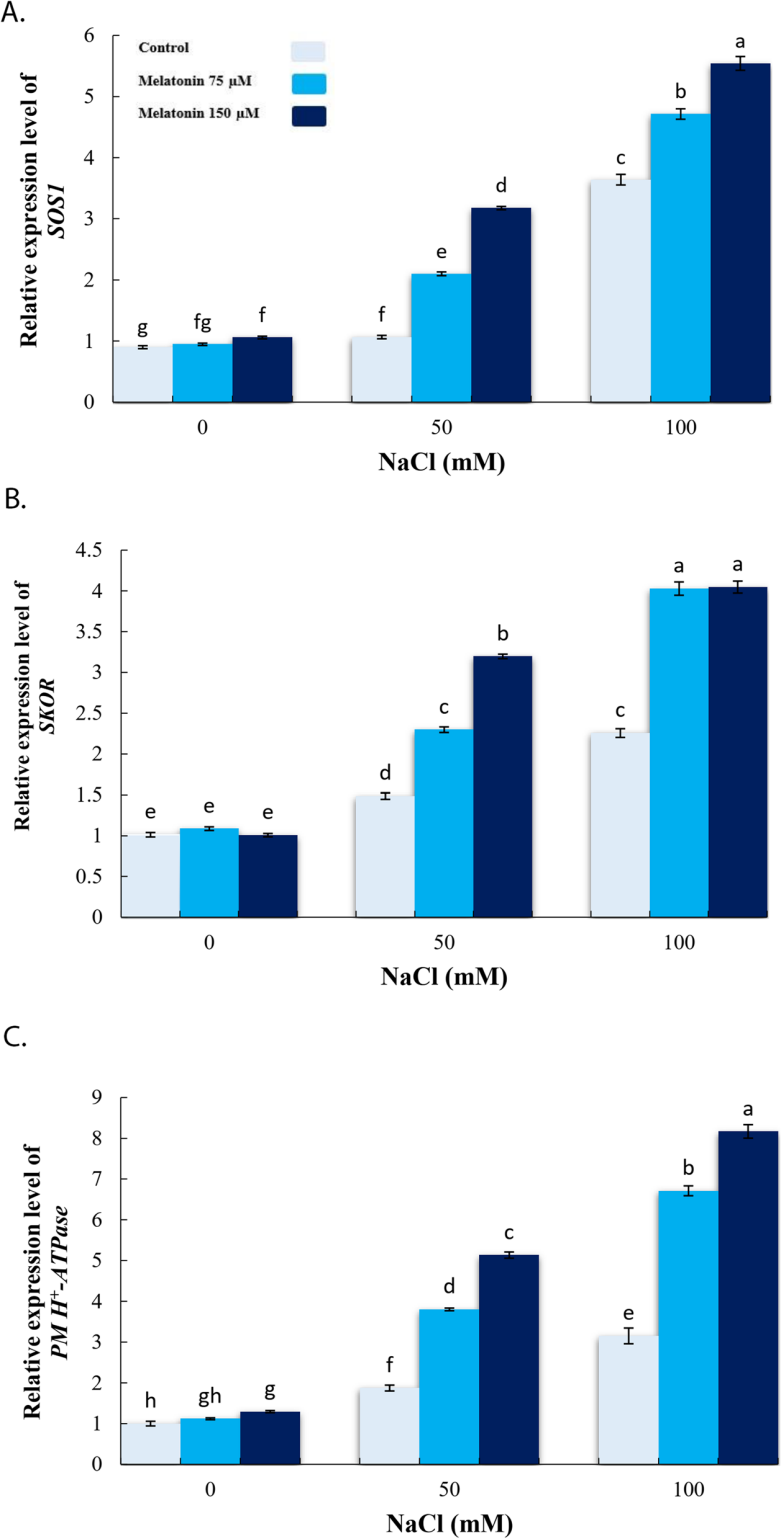
Effect of application of melatonin (0, 75 and 150µM) on relative expression level of WRKY1 (**a**), SOAR1 (**b**) and Mc5PTase7 (**c**) genes in the bitter melon leaves. Data are the average of 3 replicas ± standard error. Different letters show significant difference according to Duncan’s multiple range test at *p* ≤ 0.05

**Fig. 6:**
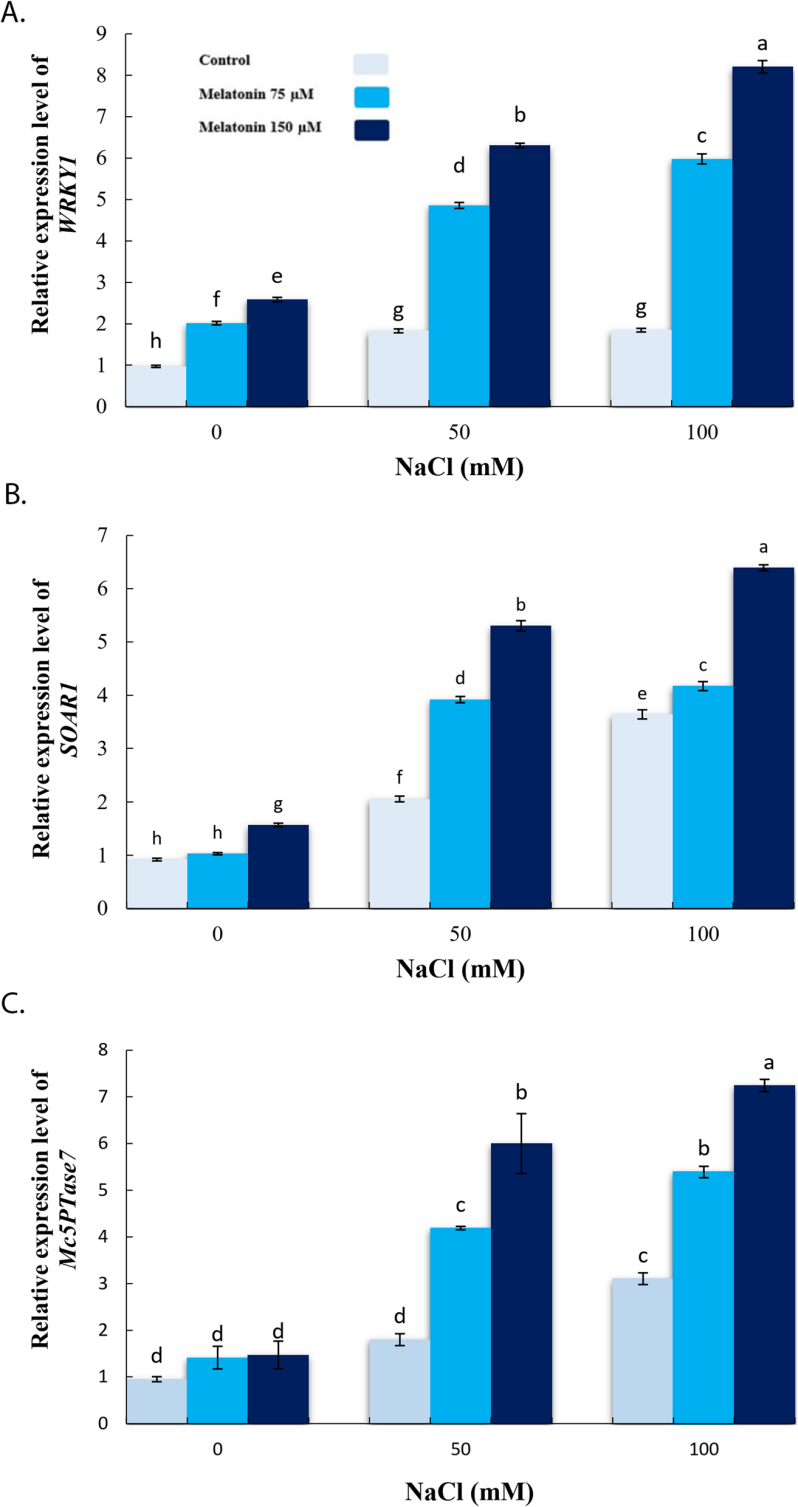
Effect of application of melatonin (0, 75 and 150µM) on relative expression level of SOS1 (**a**), SKOR (**b**) and PM H^+^-ATPase (**c**) genes in bitter melon roots. Data are the average of 3 replicas ± standard error. Different letters show significant difference according to Duncan’s multiple range test at *p* ≤ 0.05

To evaluate the effects of MT on the ion homeostasis in roots of bitter melon under salinity conditions, the transcription level of *SOS1*, *SKOR*, and *PM H*^*+*^*-ATPase* were also investigated. Accordingly, 100 mM NaCl caused significant inductions in the transcriptions level of all three genes (Fig. 5a-c), in comparison to the control. However, pre-treatment with 150 µM MT further increased expressions level of *SKOR, SOS1*, and *PM H*^*+*^*-ATPase* in relation to 50 and 100 mM NaCl treatments (Fig. 6a-c).

**Fig. 7:**
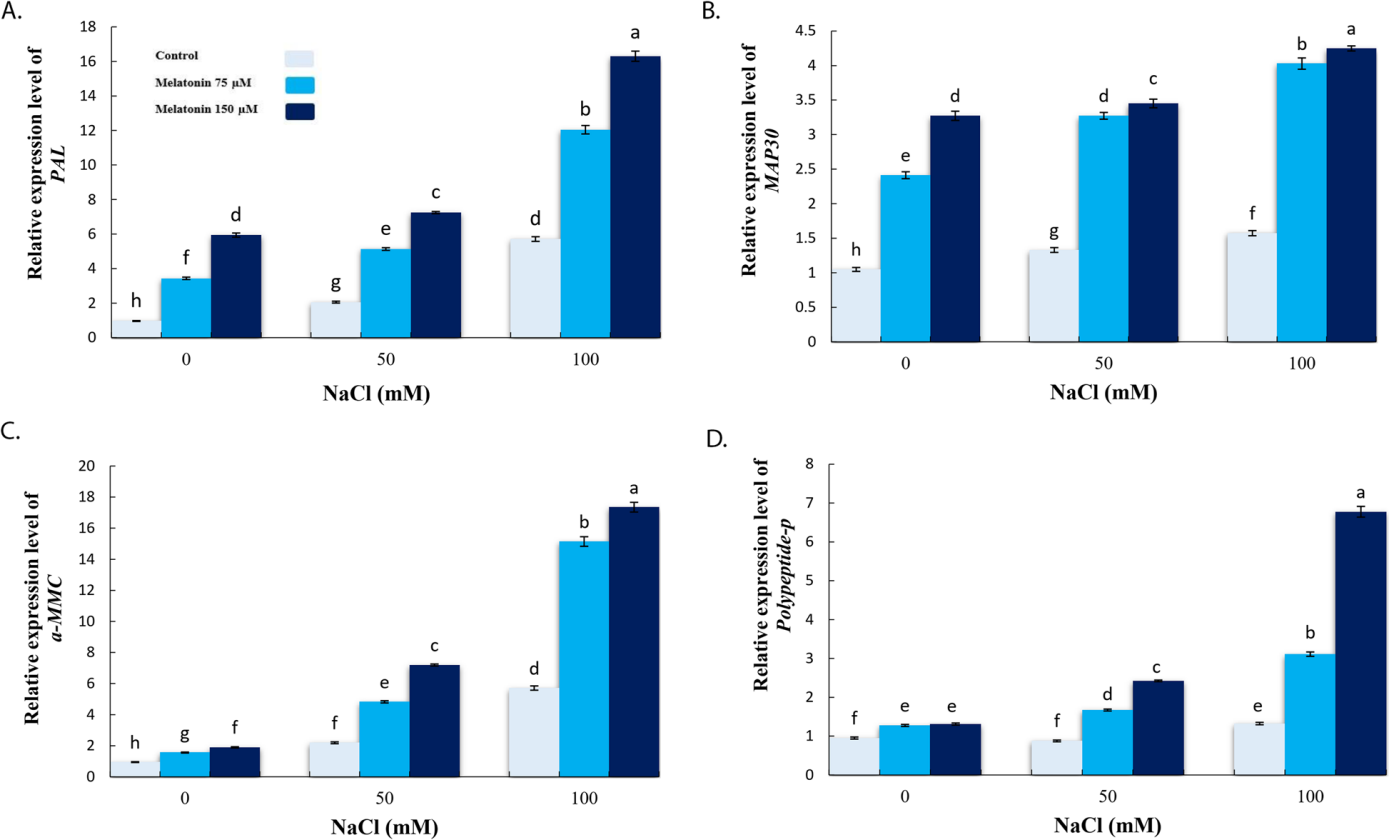
Effect of application of melatonin (0, 75 and 150µM) on relative expression level of PAL (**a**), MAP30 (**b**), a-mmc (**c**) and polypeptide-p (**d**) genes in bitter melon leaves. Data are the average of 3 replicas ± standard error. Different letters show significant difference according to Duncan’s multiple range test at *p* ≤ 0.05

Under 100 mM salinity, *WRKY1, SOAR1*, and *Mc5PTase7* were significantly up-regulated in the shoot of bitter melon plant once compared with non-saline conditions (Fig. 7a-c). However, pre-treatment with 150 µM MT showed significant up-regulation in expressions level of the three genes in shoot tissues, in comparison to the stressed plants (Fig. 5a-c).

In addition, current findings revealed that transcript levels of *PAL, MAP30, α-MMC*, and *Polypeptide-P* were significantly up-regulated under 100 mM salinity stress. As the case of other estimated parameters, pre-treatment with MT 150 µM significantly inducted PAL, MAP30, α-MMC and polypeptide-P compared with unprimed, salt-stressed plants under both NaCl concentrations (Fig. 7a-d).

## Discussion

**Fig. 8:**
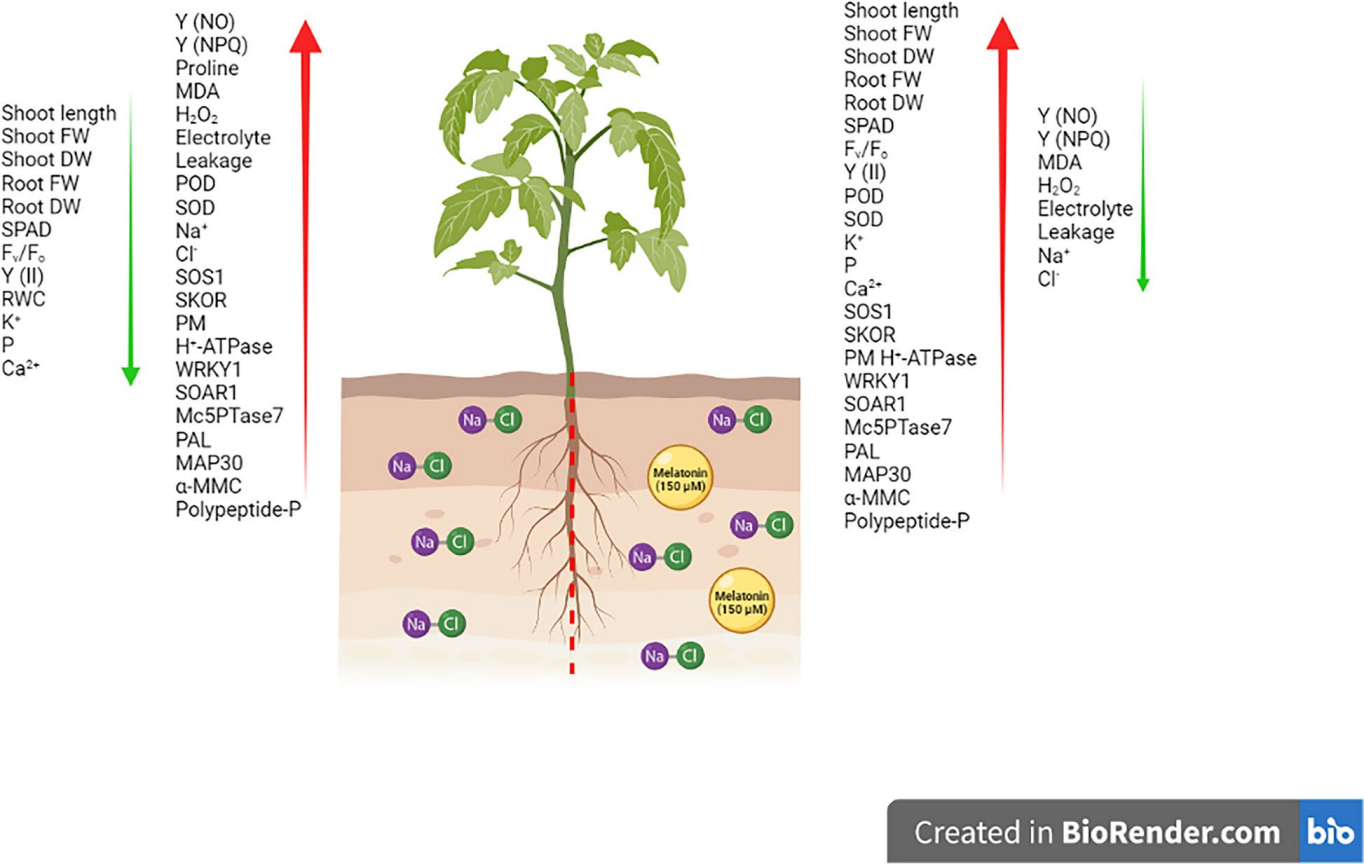
Schematic presentation of the findings of the study

In the current study, exogenous effects of melatonin on salt stress-submitted bitter melon plants were investigated through an array of agronomic, physiological and biochemical attributes. The relevant findings were collectively visualized and presented in Fig. 8. As expected, high levels of salinity critically decreased growth and photosynthetic parameters such as SPAD index, ^Fv^/_Fo_, Y(II) and increased Y(NO) and Y(NPQ). These findings are in accordance with the observations of Wu et al. [42] and Gohari et al. [43] for cucumber and Moldavian balm plants, respectively. As expected, the application of MT led to increases in SPAD index, ^Fv^/_Fo_, Y(II) and decreases in values of Y(NO) and Y(NPQ). Photosynthesis is an important process due to its pivotal role in plant survival and productivity. For that reason, reduction in photosynthesis is commonly translated into reduced growth and development. Numerous reports have shown the protective role of MT on photosynthetic apparatus of crop plants exposed to salt stress. For instance, employing MT significantly increased chlorophyll pigments, carotenoids concentration and Fm, ^Fv^/_Fm_, ETR, Y(II), and qP in cucumber plants [44]. Similarly, MT priming increased photosynthetic quantum yield (φPSII), the total content of chlorophyll, as well as *RbcL* and *RbcS* genes expression in *Phaseolus vulgaris* L. [45], while relative chlorophyll content and genes involved in photosynthesis (including *ATPF0A, ATPF0B, ATPF1B* and *LHCB*) genes were induced in melatonin-primed rubber tree (*Hevea brasiliensis*) grown under salt stress [46]. In addition, Xie et al. [47] reported that MT decreased Y(NO) and Y(NPQ) in tomato seedlings under calcium nitrate stress.

As one of the adopted strategies for combating the stress, plants accumulate osmotic regulators for maintaining intra-cellular stability and protecting their cells from the toxicity of salt stress [48]. Ferchichi et al. [49] reported that proline presents multiple roles such as regulation of salt stress-responsive gene expression, redox homeostasis, as well as stabilization of membrane and proteins. In the present experimental setup, the application of MT significantly improved proline’s concentration and RWC in bitter melons plant during salt stress, as the cases observed in several other plant species treated with MT and salt stress [50, 51]. Specifically, MT amplified proline, total soluble carbohydrate content as well as pyrroline-5-carboxylate synthase (P5CS) activity in tomatoes [52]. Furthermore, Chen et al. [50] reported that soluble sugar and soluble protein contents in cotton seeds were enhanced following MT application under salinity stress.

Current findings showed that 100 mM NaCl caused critical increments in levels of free radicals (H_2_O_2_), MDA, and relative conductivity. These findings are similar with the observations of Zhang et al. [23]. In the same way, Chen et al. [53] and Li et al. [54] reported that MT generally protects the crop plants from oxidative-induced detrimental stress by detoxification of the ROS and owing to increased activities of antioxidative enzymes. Similarly, findings of the present study revealed that MT application lowered free radicals (H_2_O_2_), MDA content and relative conductivity level by increasing activities of POD and SOD enzymes. In agreement with our findings, the application of MT increased antioxidative enzyme activities and transcriptions level of the associated genes that encode the antioxidative enzyme expressions, while decreasing free radicals (H_2_O_2_, O_2_^⋅−^), MDA, and relative conductivity in cucumber [44] and *Phaseolus vulgaris* L. [45] under salinity stress.

Maintaining ionic homeostasis in plant tissues has been linked with the status of the antioxidant enzymes, membrane integrity and osmotic potential of the cells, affecting cellular turgor which is translated into plant growth and development. In this regard, Shabala and Cuin [55] reported that maintaining a high K^+^/Na^+^ ratio and a low cytosolic Na^+^ content were essential factors for plants to maintain homeostasis of their cellular metabolism as Na^+^ and Cl^−^ were metabolically toxic at high concentrations [56]. Kurusu et al. [57] reported that Ca^2+^ is a key signaling component in a plant’s salt stress response. It has been reported that Ca^2+^ reduces the negative effects of salt stress in plants [58] by stabilizing cell wall structures [59], maintaining functional and structural integrities of membrane in plants [58], regulating ion selectivity and transport and controlling ion-exchange behavior [60]. Stressors such as cold shock [61], heat shock [62], salinity [63] and drought [61] induce cytosolic Ca^2+^ accumulation, which acts as in the form of secondary messenger during the stressful conditions signaling [64].

The SOS2-SOS3 complex activities SOS1 (Na^+^/H^+^ anti-porter), which in turn regulates cytosolic Na^+^ concentration [65]. It has been reported that SOS2 affects CAX1, thus further connecting cell Ca^2+^ with Na^+^ transportation [66]. Phosphorus (P) is an essential element of the macro-category that is involved in a variety of processes in the plants such as transfer of the energy where it is required, photosynthesis, respiration, signaling transduction cascades, and macromolecular biosynthesis [67]. The availability of P can affect salt tolerance of plants [68]. Current results showed that salt stress-imposition enhanced the Na^+^ and Cl^−^ and reduced K^+^, Ca^2+^ and P, while application of MT significantly increased K^+^, Ca^2+^ and P contents and substantially reduced Na^+^ and Cl^−^ in roots of bitter melon under salinity stress. Furthermore, MT increased *SKOR, SOS1*, and *PM H*^*+*^*-ATPases* transcripts level in the root cells of salt-stressed bitter melons in comparison to either Unprimed or salt-stressed plants. Application of MT increased transcription levels of SOS pathway genes (*SOS1-3*) in cucumber [44], PM H^+^–ATPases activities, and the homeostasis of K^+^/Na^+^ in the seedlings of sweet potatoes [69], genes expression associated with key potassium channels and transporters (*OsHAK1, OsAKT1, OsGORK* and *OsHAK5*) and K^+^ content [70], the content of Ca^2+^ [71] and reduced Na^+^ and Cl^−^ [50] under salt stress. Regarding current findings, MT significantly induced *WRKY1*, *SOAR1* and *Mc5PTase7* expression under salinity stress. The *WRKY1* transcription factor is a key component of stress-related signal transduction pathways and is a factor in the improvement of plant tolerance to stress [72]. A wide range of downstream genes [72] including jasmonic acid-responsive genes [73], and genes associated with signal transduction of salicylic acid [74] and regulators of secondary metabolism are controlled by WRKY1 [72]. In agreement with our findings, MT induced up-regulation of various genes expression such as *MYB*, *WRKY*, and other (genes) transcription in Arabidopsis and cucumber [75, 76] and *DREB*, *WRKY*, and *MYB* in Bermuda grass [77].

SOAR1 is a downstream of the ABA receptor and upstream of an important ABA-responsive bZIP transcription factor [11]. Ma et al. [78] stated that two isoforms of Arabidopsis eIF4G, eIFiso4G1 and eIFiso4G2 interacted with SOAR1 in order to regulate ABA signaling negatively. In addition, Bi et al. [79] reported that both *USB1* and *SOAR1* were required genes for transcripts splicing of numerous genes such as the genes associated with salinity responses and signaling of ABA pathways. The over-expression of *SOAR1* also increased proline levels, expression levels of *SOS1, SOS2*, and *P5CS1* and growth of plants under salinity while it decreased the level of electrolyte leakage [10]. Our results showed that MT increased growth, transcript levels of *SOAR1* and *SOS1*, proline, and decreased electrolyte leakage under salinity stress conditions.

Huang et al. [7] reported that certain ROS production has been found associated with NADPH oxidase-which is further linked with tolerance of salinity in different plants. The main apoplastic ROS generation source is burst oxidase homolog (RBOH). Torres et al. [80] described that RBOH generated superoxide, which was then dismutated to H_2_O_2_ [81].

Kaye et al. [82] found that *AtRbohJ* plays a key role in production of ROS in plants under salinity stress and the production of ROS in *AtRbohJ* mutants was significantly lower under salinity stress. Other reports revealed that the transcriptions of *AtRbohJ* in *At5ptase7* mutants were significantly decreased during salt stress [82]. Those findings suggest that *At5ptase7* plays an imperative function in production of ROS and NADPH oxidase activity under salinity stress. It was observed that *At5PTase7* mutants showed failure in the induction of *RD22* and *RD29A*, that contains numerous ROS-reliant components with regard to their certain promoters [82]. NADPH oxidase activity increased rosette fresh weight, K^+^ concentration, K^+^/Na^+^ ration, total chlorophyll content, chlorophyll fluorescence (^Fv^/_Fm_), CAT, APX, GR and SOD enzymatic activities, and decreased Na^+^, H_2_O_2_ and MDA concentrations in *Arabidopsis thaliana* under salt stress [83]. Present findings showed that application of MT increased transcript levels of *Mc5PTase7*, fresh weight, K^+^ concentration, SPAD, ^Fv^/_Fo_, Y(II), POD and SOD enzymatic activities, and decreased Na_+_, H_2_O_2_ and MDA concentrations in salt-stressed plants. Similar to our conclusion, Chen et al. [84] reported that MT may improve tolerance against certain stresses through modulation of ROS-signaling which is well co-ordinated by NADPH oxidase.

Several important proteins and peptides such as 30 kD (MAP-30) which is an anti-HIV protein, α-momorcharin (α-MMC) and polypeptide-P were isolated from bitter melon. MAP30 and α-MMC is a single chain RIP (type I ribosome-inactivating proteins) and their molecular mass are 30 kD. MAP30 and α-MMC prevent many types of cancers such as blood, brain, breast, colon, liver, and lung cancer [85]. In addition, polypeptide-P, a hypoglycemic peptide, has an imperative function in the recognition of cells and certain reactions required for the adhesion purpose [30]. In this study, the application of MT increased *MAP30*, *α-MMC*, *polypeptide-P* and *PAL* gene expression levels under control and saline conditions. The current molecular profiles are in accordance with earlier reports that MT treatment positively induced transcription of flavonoid biosynthetic genes such as *C4H*, *PAL, LAR, CHS, F3H, ANR*, and *UFGT* in kiwifruit [86], genes related to the biosynthesis of rosmarinic acid (*PAL* and *RAS*) in *Dracocephalum kotschyi* [87], phenylpropanoid pathway genes (*PAL, STS*) in grape berries [88] and biosynthesis associated genes of anthocyanin (*C4H, PAL, CHI, CHS, DFR, LDOX, F3H, F3′H, GST and UFGT*) in red cabbage and white cabbage [27].

## Conclusions

Exogenous MT enhanced salinity stress tolerance in bitter melon through different mechanisms. Exogenously applied MT (150 µM) in salt-stressed plants improved growth and photosynthetic parameters, increased osmoprotectant through higher proline content, lowered oxidative stress by up-regulating antioxidant enzymatic activity, regulated ionic homeostasis and importantly, resulted in the transcriptional regulation of multiple defense-related genes. Furthermore, MT induced the transcription levels of genes linked to the secondary metabolites. Overall, it can be concluded that MT can be successfully employed as an effective priming agent for the amelioration of salt stress in bitter melon plants.

## Supplementary Information

**Additional file 1.**

## Data Availability

The datasets used and/or analyzed during the current study are available from the corresponding author on reasonable request.
