# Supplementary material for: Exogenous melatonin increases salt tolerance in bitter melon by regulating ionic balance, antioxidant system and secondary metabolism-related genes
Source: BMC Plant Biol. 2022 Jul 30;22:380. doi: 10.1186/s12870-022-03728-0 (PMC9338570; doi:10.1186/s12870-022-03728-0)
Supplement: Supplementary file 1 — Additional file 1. [file 12870_2022_3728_MOESM1_ESM.docx]

**Table S1.** Primer sequences for examined genes.

| Reference | Forward (5’—3’)  Reverse (5’—3’) | Gene |
| --- | --- | --- |
|  | GTTCTCTCATTTCTAATTCTCGCA  GTTTTTCCGTCGTAATTGAAGAG | *a-MMC* |
|  | GGAAAATCAATGGTCTGCTCTCT  ATTCCCCAAGTAGAGTCATGGT | *MAP30* |
|  | CGTGATGAAGGCAAAGTGGATGA  ACGCATGTTTCAGTTCCGCAG | *Polypeptide-P* |
|  | TCGTCGCTGTTCTCTGTTTGTG  CACCAACAACCCCAAATAGCATAA | *SOS1* |
|  | ATTCGTTGGTTTGTTGCCCCTT  GCATCGTTCACACCATCTCCT | *PM H+-ATPase* |
|  | ATGGATAAGGCTATTTCGGGT  CAAAGATGTGGTGTAGCGT | *SKOR* |
|  | GCAATTCTCCTCTTCGTCAGG  CCTGACGAAGAGGAGAATTGC | *SOAR1* |
|  | CCTACCGGCATAAGGAGCAGAT  TTTGCTGGAAGATGGAGCAA | *Mc5PTase7* |
| Rajaee Behbahani et al., (2020) | AGTGATGGCATAGTCCTCGAT  CCAGGAATATGCGAACTTAGCTT | *WRKY1* |
| Rajaee Behbahani et al., (2020) | ATTGGGAAGCTCATGTTTGC  GGTGACGGGATTTGCTAAGA | *PAL* |
|  | AGTGTTCTCTCAACCCACTC  AGCATGAAGTGGATTCTTGG | *α-tubulin* |
